# Surface Quality Improvement of 3D Microstructures Fabricated by Micro-EDM with a Composite 3D Microelectrode

**DOI:** 10.3390/mi11090868

**Published:** 2020-09-19

**Authors:** Jianguo Lei, Kai Jiang, Xiaoyu Wu, Hang Zhao, Bin Xu

**Affiliations:** Guangdong Provincial Key Laboratory of Micro/Nano Optomechatronics Engineering, Shenzhen University, Shenzhen 518060, China; ljg_sc111@163.com (J.L.); 13728644743@163.com (K.J.); wuxy@szu.edu.cn (X.W.); zh@szu.edu.cn (H.Z.)

**Keywords:** micro-EDM, composite 3D microelectrode, diffusion bonding, step, 3D microstructure

## Abstract

Three-dimensional (3D) microelectrodes used for processing 3D microstructures in micro-electrical discharge machining (micro-EDM) can be readily prepared by laminated object manufacturing (LOM). However, the microelectrode surface always appears with steps due to the theoretical error of LOM, significantly reducing the surface quality of 3D microstructures machined by micro-EDM with the microelectrode. To address the problem above, this paper proposes a filling method to fabricate a composite 3D microelectrode and applies it in micro-EDM for processing 3D microstructures without steps. The effect of bonding temperature and Sn film thickness on the steps is investigated in detail. Meanwhile, the distribution of Cu and Sn elements in the matrix and the steps is analyzed by the energy dispersive X-ray spectrometer. Experimental results show that when the Sn layer thickness on the interface is 8 μm, 15 h after heat preservation under 950 °C, the composite 3D microelectrodes without the steps on the surface were successfully fabricated, while Sn and Cu elements were evenly distributed in the microelectrodes. Finally, the composite 3D microelectrodes were applied in micro-EDM. Furthermore, 3D microstructures without steps on the surface were obtained. This study verifies the feasibility of machining 3D microstructures without steps by micro-EDM with a composite 3D microelectrode fabricated via the proposed method.

## 1. Introduction

Micro electrical discharge machining (micro-EDM), as a non-conventional machining technology, possesses unique advantages, such as non-contact machining nature and negligible cutting force, and is capable of machining any electrically conductive materials regardless of the hardness. Thus, micro-EDM was widely used to machine automotive, aerospace and surgical microstructures [1,2,3,4].

For the fabrication of three-dimensional (3D) microstructures, the current micro-EDM technology usually utilizes a layer-by-layer milling process with a cylindrical microelectrode. Yu et al. [5] developed a uniform wear method (UWM) for 3D micro-EDM. Based on the UWM, various complicated 3D microstructures were successfully fabricated via layer-by-layer scanning micro-EDM with a cylindrical electrode. Reynaerts et al. [6] fabricated a 3D microstructure consisting of two planes inclined under 45° in silicon by EDM milling, and found that the EDM process was independent of the silicon crystal orientation. Rajurkar et al. [7] integrated computer aided design and manufacturing (CAD/CAM) systems with micro-EDM, while accounting for tool wear utilizing UWM, and successfully generated various complex 3D micro cavities. Bleys et al. [8,9] introduced improved wear compensation and real-time wear sensing based on discharge pulse evaluation in EDM milling, achieving the accurate machining of square and hexagonal pockets. Zhao et al. [10] applied CAD/CAM system in micro-EDM to improve the machining quality, and successfully prepared a human face embossment with a dimension of 1 mm × 0.4 mm using a 30 μm diameter copper rod electrode.

To improve the machining accuracy and efficiency, based on the scanned area in each layer machining, Li et al. [11] proposed a new compensation method, which was integrated with a CAD/CAM system in 3D micro-EDM milling to generate 3D micro cavities. According to the proportional relationship between the removed workpiece volume and the number of discharge pulses, Jung et al. [12] developed a control method for a micro-EDM process using discharge pulse counting, improving the machining efficiency and accuracy without complex path planning to compensate electrode wear. Using a machine vision system, Yan et al. [13] proposed a multi-cut process planning method and an electrode wear compensation method for layer-by-layer 3D micro-EDM. Various 3D microstructures, such as pyramid cavity, hexagonal pyramid cavity, rectangular pyramid, and cone cavity, were prepared. This approach significantly improved machining accuracy and reduced machining time.

To improve the machining stability and the effective discharge ratio, Tong et al. [14] proposed workpiece vibration-assisted servo scanning 3D micro-EDM. With the optimized parameters, a series of 3D microstructures such as double-prism cavity with hemisphere, round cavity with hemisphere, camber and rectangular pyramid were prepared. To improve the machining surface quality and reduce electrode wear, Song et al. [15] developed a spray EDM milling method with a bipolar pulsed power source and deionized water. To improve the machining performance, such as material removal rate, electrode wear ratio and surface roughness, Yu et al. [16] combined the linear compensation method and the UWM to machine 3D microstructures in micro-EDM. Bissacco et al. [17,18] proposed a new method based on the discharge counting and discharge population characterization, effectively compensating tool electrode wear in micro-EDM milling.

Based on the established mathematical model of erosion depth, Li et al. [19] proposed a strategy of scanning speed adjusted with layer in 3D micro-EDM milling, effectively lowering the accumulative depth error to less than 1% under appropriate conditions. Tong et al. [20] proposed an on-machine process of rough-and-finishing servo scanning 3D micro-EDM to machine 3D micro cavities. In the rough machining process, high discharge energy and large-diameter tool electrodes were used to improve processing efficiency. In the finishing machining process, a small amount of material was removed by changing multi-factors of machining parameters. Based on the discharge pulse behavior, Wang et al. [21] combined off-line and in-line adaptive tool wear compensation to achieve effective and efficient tool wear compensation in micro-EDM milling, and prepared hemisphere, cone and pyramid cavities, with good form accuracy on the stainless steel. Tong et al. [22] developed a novel process of 3D servo scanning micro-EDM with the movement of two-axis linkage and one-axis servo, and efficiently and cheaply machined 3D complex micro driving structures pierced through thin-walled micro tube of NiTi SMA. D’Urso et al. [23] fabricated a micro-pocket on zirconium carbide ceramics by micro-EDM milling using a tungsten carbide cylindrical electrode of diameter 0.3 mm.

Micro-EDM milling simplifies the 3D machining into two-dimensional (2D) laminated scanning, however, the machining time is still long [5,10], which is mainly caused by the smaller cross section of the microelectrode and the milling strategy. To improve the anti-interference ability of the microelectrode, Xu et al. [24] introduced a foil queue microelectrode in micro-EDM to fabricate a 3D microstructure. The impacts of machining voltage and pulse width on rounded corner wear at the end of the foil microelectrode and the step effect on the 3D microstructure surface were investigated. The 3D microstructures with hemispherical shapes were successfully machined, and the dimensional errors of the 3D microstructures were less than 10 µm. Using the difference in the wear rates of different materials in EDM, Lei et al. [25] fabricated surface microstructures on Ti–6Al–4V alloy workpieces by EDM with laminated disc electrodes made of Cu and Sn foils, converting disadvantageous phenomenon of wear into a beneficial process. There was physical contact without metallurgical reaction between the foils. To improve the machining efficiency, based on the laminated object manufacturing (LOM), Xu et al. [26] fabricated a laminated 3D microelectrode with a complicated contour and shape via combining wire electrical discharge machining (WEDM) and thermal diffusion welding, and used the 3D microelectrode stacked by Cu foils to manufacture 3D microstructures by an up-and-down reciprocating machining method. To improve the machining accuracy, Xu et al. [27] prepared a 3D queue microelectrode by WEDM and vacuum pressure thermal diffusion welding, and applied it in micro-EDM to machine 3D microstructures. However, numerous and obvious ridges emerged on the machining surface of the 3D microstructure duo to imperfect thermal diffusion bonding between the Cu foils. To eliminate ridges on the machining surface, using Cu foils coated with Sn film as laminated materials, Lei et al. [28] manufactured laminated composite 3D microelectrodes by femtosecond laser cutting using a bending-and-avoidance mode and transient liquid phase bonding. Moreover, 3D microstructures without ridges on the surface were successfully machined by up-and-down reciprocating micro-EDM with the composite 3D microelectrodes.

LOM, as an additive manufacturing technology, is capable of fabricating various complex 3D functional micro parts. Without doubt, the LOM technology is suitable for preparing complex 3D microelectrodes, which are difficult to fabricate by traditional machining methods. However, when the 3D microelectrode possesses an inclined plane or curved surface, steps inevitably occur on the 3D microelectrode surfaces, due to the theoretical errors of LOM [29], and the steps are copied to the 3D microstructure surface on the workpiece.

To solve the problem above, the paper proposed a filling method to eliminate the steps on the 3D microelectrode surface. First, femtosecond laser was used for cutting Cu foils coated with Sn to obtain 2D layers. Then, 3D microelectrodes without steps were fabricated by applying the layers to thermal diffusion bonding, through which they were bonded and partially melt-softened (Cu) on the interface fills in the steps. Finally, 3D microstructures with no steps were successfully fabricated by micro-EDM using these electrodes.

## 2. Experimental Details

### 2.1. Experimental Setup

The main experimental setup for fabricating 3D microelectrodes and performing micro-EDM consists of a femtosecond laser, a PI motion platform, a high frequency pulse power supply, an oscilloscope, a control system and a vacuum furnace.

**Figure 1:**
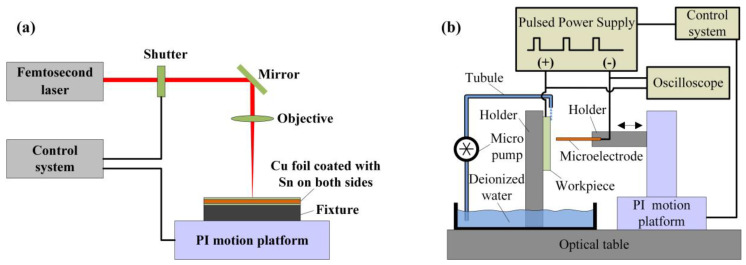
Schematic diagram of experimental setup: (**a**) Femtosecond laser cutting system; (**b**) Micro- electrical discharge machining (micro-EDM) system.

The titanium sapphire femtosecond laser, with a central wavelength of 800 ns, a pulse duration of 35 fs, a maximum repetition frequency of 1 kHz and pulse energy of 4 mJ, from Coherent Inc. was used to cut Cu foils coated with and without Sn to obtain 2D microstructures (Figure 1a) [30]. The high precision motion platform (model: M511.DD) made by Germany, PI was used to control the femtosecond laser cutting path and perform micro-EDM experiments. The maximum strokes of X/Y/*Z*-axis were 100 mm and the motion accuracy of each axis was of 0.2 μm. A wire EDM machine manufactured by HI-LINK Precision Machinery Co., Ltd., (Shenzhen, China) (model: H-CUT 32F), was used on fabricated samples for observation and characterization. The maximum stroke of *X*-axis, *Y*-axis and *Z*-axis were 400 mm, 320 mm and 400 mm, respectively. The location precision was 1 μm for all axes.

**Figure 2:**
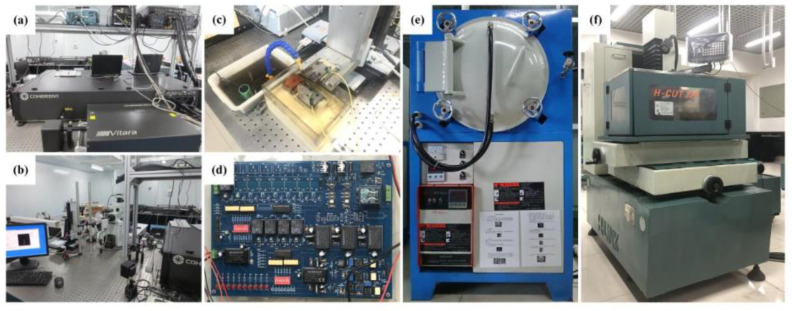
Pictures of experimental setup: (**a**,**b**) Femtosecond laser cutting system; (**c**) Micro-EDM platform; (**d**) Pulse power supply; (**e**) Vacuum furnace; (**f**) Wire EDM machine.

The schematic diagram of micro-EDM setup is shown in Figure 1b. A laminated 3D microelectrode was fixed on the PI platform to machine microstructures through a back and forth reciprocating machining strategy. The high frequency pulse power supply was used to generate ultra-short pulse signal and the machining process was real-time monitored using the oscilloscope. The pictures of experimental setup are displayed in Figure 2a–f.

### 2.2. Experimental Materials and Conditions

Here, 50-μm-thick Cu foils coated with Sn on both sides were utilized to fabricate 3D microelectrodes. The thicknesses of Sn were 0 μm, 0.5 μm, 2.0 μm, and 4.0 μm, respectively. That is, the thickness of Sn layer in the sandwich structure was 0 μm, 1 μm, 4 μm, and 8 μm, respectively. Notably, 1 mm thick #304 stainless steel was used as a workpiece to machine microstructures. The working fluid used in the micro-EDM process was deionized water. According to the previous study [26,27,28], the machining conditions are selected to prepare the 3D microelectrodes and microstructures, as listed in Table 1.

### 2.3. Measurement and Evaluation

For observation and characterization, the fabricated 3D microelectrodes and the machined microstructures were cut by wire EDM and were polished afterwards. The morphology of the samples was observed by scanning electron microscopy (SEM) (S-3400N, Hitachi, Co., Ltd., Tokyo, Japan). To analyze the distribution of Cu and Sn in the steps and the matrix, energy dispersive X-ray spectrometer (EDS) point analysis was employed. The wear of the 3D microelectrodes and the surface roughness of the machined microstructures were measured by a laser scanning confocal microscope (VK-X250, KEYENCE, Osaka, Japan).

## 3. Results and Discussion

### 3.1. Fabrication of the Laminated 3D Microelectrode and the Microstructure

**Figure 3:**
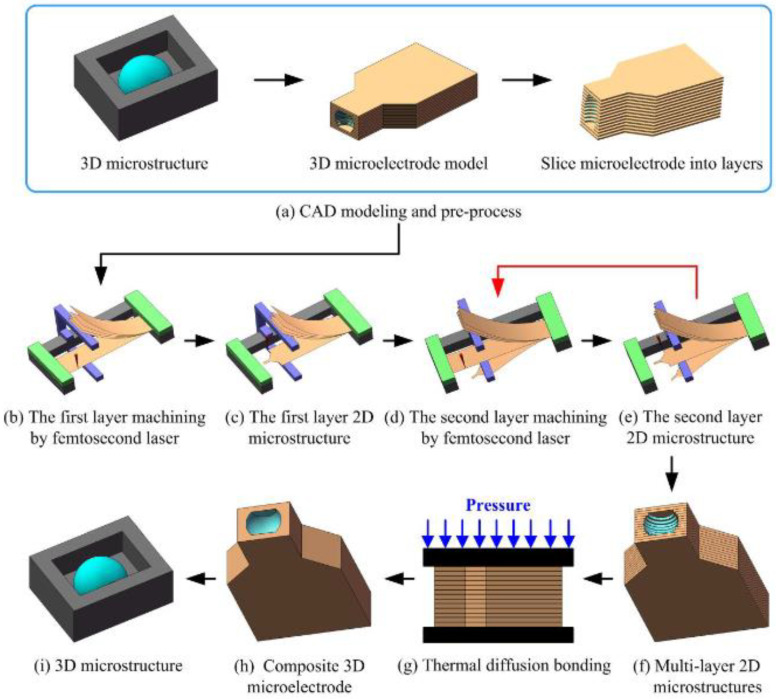
Schematic diagram for fabricating 3D microelectrode and microstructure.

Based on the LOM technology, the process of fabricating 3D microelectrodes and microstructures is generally performed by the following steps:

(1) CAD modeling and pre-process. Based on a 3D microstructure model, a 3D microelectrode model was prepared. Then, the 3D microelectrode model was sliced into several thin cross-sectional layers in a given direction by slicing software. After that, the total layer number and the profile data of each 2D cross-sectional layer were obtained, as shown in Figure 3a.
(2) Femtosecond laser cutting. One end of the same multi-layer Cu foils coated with Sn on the both sides was clamped on a fixture, and the other end of the first layer was fixed on the fixture by a magnet. The other layers of the Cu foils coated with Sn were bent upward with a stopper to leave enough space for femtosecond laser cutting (Figure 3b). After completion of the first layer cutting (Figure 3c), it was bent downward with the stopper, and the former upward end of the second layer was fixed on the fixture (Figure 3d). With the above steps repeated (Figure 3d,e), multi-layer 2D microstructures were obtained (Figure 3f).
(3) Diffusion bonding. The obtained multi-layer 2D microstructures were first cleaned using ethanol in an ultrasonic bath to remove contamination and dried with nitrogen at room temperature. After cleaning, they were sandwiched between two graphite blocks and placed in a vacuum furnace, where they were pressed by a constant pressure (36 KPa). Diffusion bonding was finally carried out at the required temperature at the rate of 20 °C/min to obtain 3D microelectrodes (Figure 3g,h).
(4) Micro-EDM. The obtained 3D microelectrode was installed on the micro-EDM platform to machine 3D microstructures. The obtained 3D microstructure is shown in Figure 3i.

### 3.2. Bonding Temperature

**Figure 4:**
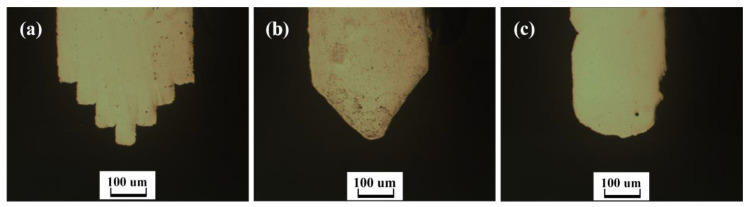
Sectional views of microelectrodes fabricated with different bonding temperatures: (**a**) 900 °C; (**b**) 950 °C; (**c**) 1000 °C.

**Figure 5:**
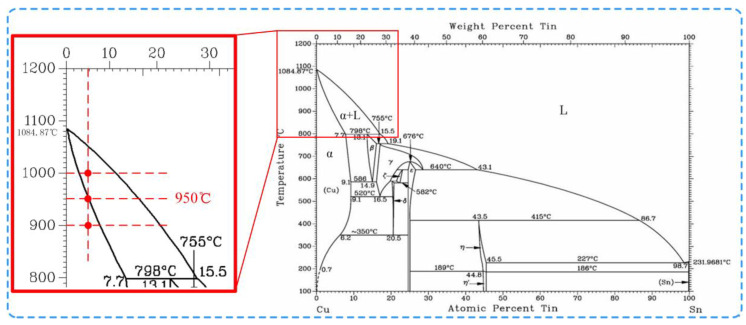
Cu-Sn phase diagram [30].

Bonding temperature plays a great role on Cu-Sn interface reaction and the component of the compounds. To fully eliminate the steps on the surface of the 3D microelectrodes, Cu foils coated with 4-μm-thick Sn on both sides were chosen to fabricate V-shaped 3D microelectrodes. Figure 4 shows the cross-section profile of the V-shaped microelectrodes when bonding time is 15 h, bonding temperature is 900 °C, 950 °C and 1000 °C respectively. The bonding time of 15 h can guarantee that Sn and Cu atoms diffuse adequately [28]. It can be seen that, when bonding temperature was 900 °C, the steps were obvious, and no interface compounds was extruded at the steps (Figure 4a). EDS point analysis was carried out on the interface, which showed a content of nearly 5 wt.% Sn. Cu-Sn phase diagram (Figure 5) presented (Cu) was still in solid state under this temperature. Therefore, under constant pressure could not be squeezed into the steps. While temperature increased to 950 °C, the steps disappeared (Figure 4b). On the interface and the steps, the content of Sn was still around 5 wt.%, at which interface compound was on solid phase line (α + L), namely the critical melting point. Under the action of a constant pressure of 36 KPa, the interface compound (Cu) was gradually squeezed to the steps and after 15 h full diffusion reaction, thus V-shaped microelectrodes were with no steps. As the temperature further went up to 1000 °C, interface compounds crossed the line and were totally in phase (α + L), featured by rheological behavior. With pressure working together, V-shaped microelectrodes were badly deformed (Figure 4c), which explained the reason that 950 °C was the optimized temperature.

### 3.3. Thickness of the Sn Film

To study the effect of Sn layer thickness on the steps, Cu foils (50 μm in thickness) coated with Sn film in different thickness were chosen to machine V-shaped microelectrodes. Then, the prepared microelectrodes were carried out micro-EDM to fabricate V-shaped microstructures on a #304 stainless steel workpiece. The process parameters were: machining voltage of 90 V, pulse width of 800 ns, pulse interval 4200 ns.

**Figure 6:**
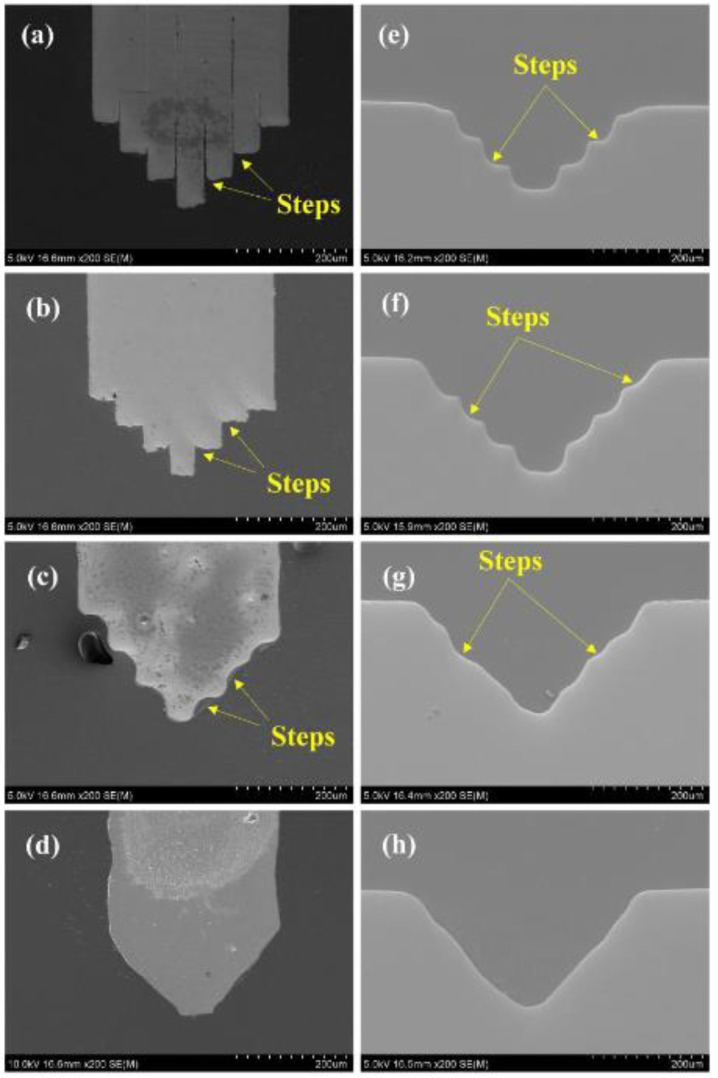
Sectional views of microelectrodes fabricated with different Sn layer thickness: (**a**) 0 μm; (**b**) 1 μm; (**c**) 4 μm; (**d**) 8 μm; (**e**–**h**) Corresponding sectional views of microstructures machined with these microelectrodes.

When the thickness of Sn layer was 0 μm, the steps were obvious on the interface, bonding quality between Cu foils was poor (Figure 6a), and the microstructures machined using this microelectrode had clear steps on the surface (Figure 6e). As the thickness of Sn layer grew to 1 μm, though with a much better bonding of the Cu foils, the steps existed on the microelectrodes (Figure 6b) and were further copied to microstructures (Figure 6f). While the thickness of Sn continued to increase, partially melt-softened (Cu) was extruded out and flowed to the steps. After 15 h full diffusion reaction, the steps on the surface of the V-shaped microelectrodes decreased (Figure 6c,d) and so did those on the corresponding 3D microstructures (Figure 6g,h). However, on the other hand, if the Sn layer is too thick, the extruded (Cu) would be beyond the capacity of the steps, decreasing the quality of the microelectrodes. Therefore, as for Cu foils 50 μm thick, the optimized thickness of Sn layer on the interface would be 8 μm.

### 3.4. The Distribution of Elements

A uniform distribution of Cu and Sn elements on the 3D microelectrodes was beneficial for obtaining identical resistivity at each position of the microelectrode. With the bonding temperate of 950 °C and Sn layer thickness of 8 μm, a laminated composite 3D microelectrode was fabricated to study the distribution of Cu and Sn elements. EDS was chosen to analyze the steps and matrix of the microelectrodes.

**Figure 7:**
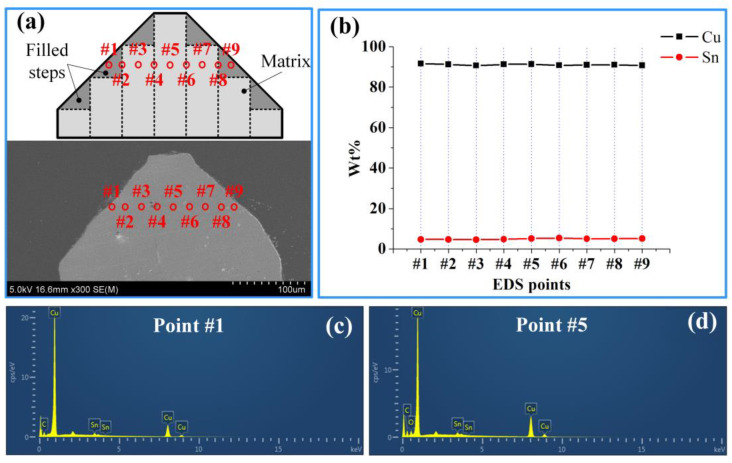
(**a**) Sectional views of microelectrodes; (**b**) The distribution of Cu and Sn along the laminated direction; (**c**) energy dispersive X-ray spectrometer (EDS) result of point #1; (**d**) EDS result of point #5.

Figure 7a illustrates section view of the V-shaped composite 3D microelectrode with no steps and EDS was carried out on point #1 to point #9. The experimental results are shown in Figure 7b. It could be seen from the diagram that all the points had uniform contributions of Cu and Sn, which indicated that after 15 h sufficient diffusion under 950 °C, Cu and Sn evenly distributed in the 3D microelectrodes. It effectively avoided the production of ridges due to the uneven distribution of Cu and Sn. Figure 7c,d respectively represent the energy spectra of point #1 and point #5 in Figure 7a. The composite 3D microelectrode mainly contained Cu and Sn. C and O may have resulted from the graphite blocks during the thermal diffusion bonding of multi-layer 2D microstructures. The content of C and O were both very low and did not affect the machining performance of the microelectrodes.

### 3.5. Fabrication of the Microstructure with a Hemisphere

**Figure 8:**
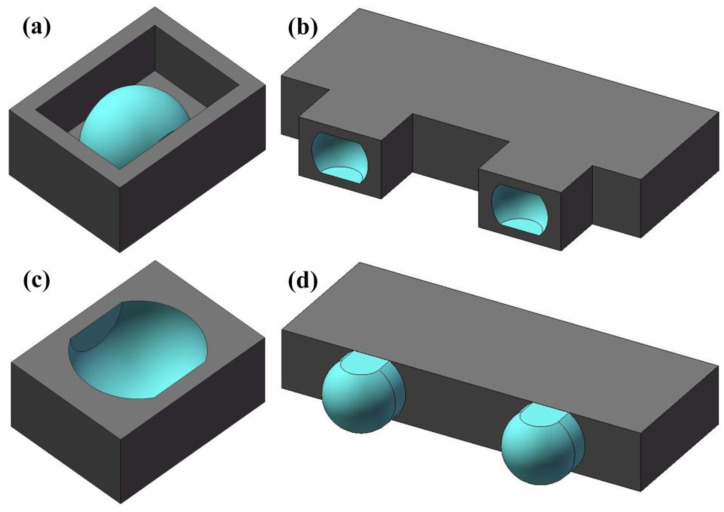
Models of (**a**,**c**) 3D microstructures and (**b**,**d**) queued 3D microelectrodes.

To verify the feasibility of the process, two kinds of 3D microstructures were designed, as shown in Figure 8a,c. It is well known that tool electrode wear inevitably significantly reduces the machining accuracy of microstructures. Therefore, the queued 3D microelectrode models were established according to the 3D microstructures (Figure 8b,d).

**Figure 9:**
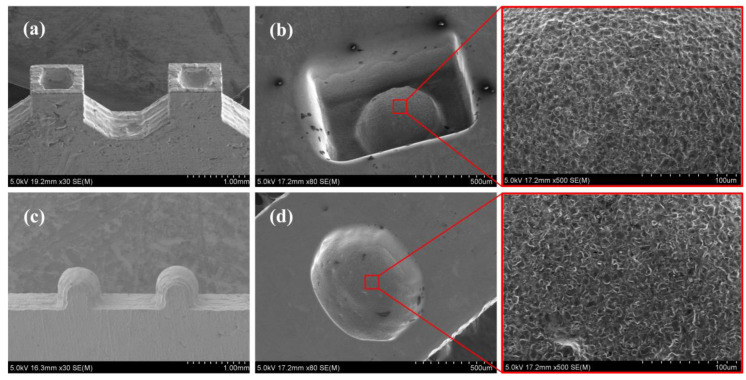
(**a**,**c**) Composite 3D microelectrodes; (**b**,**d**) 3D microstructures machined by EDM.

Notably, 50-μm-thick Cu foils coated with 4 μm thick Sn films on both sides were cut by a femtosecond laser beam to obtain multi-layer 2D microstructures. To obtain high quality 2D microstructures, the cutting parameters of femtosecond laser were a 400 mW laser power and a 100 μm/s cutting speed. The multi-layer 2D microstructures were heated up to 950 °C for 15 h with a pressure of 36 KPa in a vacuum furnace to obtain the queued 3D microelectrodes, as shown in Figure 9a,c. It can be observed that there were no steps on the surface of the 3D microelectrodes. This indicated that the steps had been filled effectively by the extruded melt-softened (Cu) during the diffusion bonding process.

To further verify the elimination of the steps on the surface of the 3D microelectrode, the prepared queued 3D microelectrodes were applied to back-and-forth micro-EDM in sequence, after which 3D microstructures were gained, as shown in Figure 9b,d. The machining parameters were set as follows: voltage 90 V, pulse width 800 ns, pulse interval 4200 ns, pulse frequency 0.2 MHz. The working fluid was deionized water. It can be seen from Figure 9b,d that the arc surface of 3D microstructures had no steps, which indicated that the steps on the 3D microelectrode surface were eliminated. By laser scanning confocal microscopy, surface roughness analyses were carried out on the arc surface of Figure 9b,d. The results were *R_a_* = 1.365 μm and *R_a_* = 1.51 μm, respectively. The use of deionized water significantly reduced microelectrode wear. The wear of the first and the second microelectrode in the queued 3D microelectrodes were 10 μm and 5 μm, respectively.

**Figure 10:**
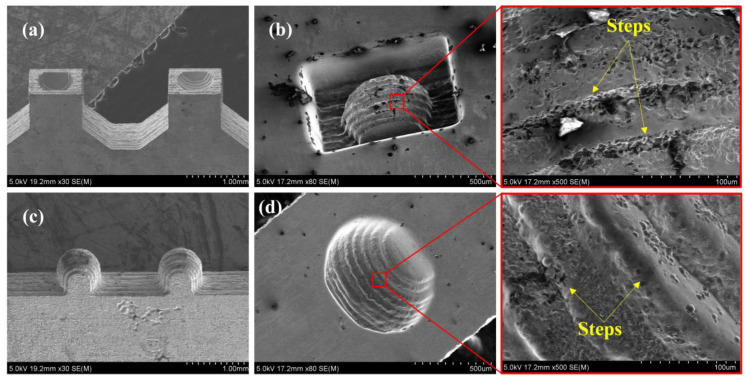
(**a**,**c**) Laminated 3D microelectrodes with steps; (**b**,**d**) Microstructures machined by micro-EDM.

For comparison, under the same process conditions, Cu foils coated with 0.5 μm thickness Sn film were used to fabricate queued 3D microelectrodes (Figure 10a,c), and the fabricated queued 3D microelectrodes were applied in micro-EDM to process 3D microstructures (Figure 10b,d). The experiment results show that the steps on the surfaces of 3D microelectrodes and microstructures were extremely obvious. It indicated that the amount of Sn on the interface was insufficient. Therefore, the steps on the surfaces of 3D microelectrodes still existed. As a result, the steps were copied to the machined surface of 3D microstructures from the 3D microelectrode surface.

## 4. Conclusions

In this paper, a filling method was proposed to eliminate the steps on the surface of laminated composite 3D microelectrodes using melt-softened (Cu). The microelectrodes were then used to machine 3D microstructures in stainless steel sheets by micro-EDM. The experimental results can be summarized as follows:

(1) The bonding temperature had a great influence on the state of Cu-Sn intermetallic compounds. The steps on the surface of laminated 3D microelectrodes were eliminated by controlling the bonding temperature to make the melt-softened (Cu) fill in the steps.
(2) When the thickness of Sn on Cu foil sides (50 μm thick) was 4 μm, bonding temperature was 950 °C and bonding time was 15 h, the composite 3D microelectrode without steps on the surface was successfully fabricated. Cu and Sn elements were uniformly distributed, both in the steps and matrix.
(3) The queued composite 3D microelectrodes fabricated with the optimized parameters were applied in micro-EDM, obtaining 3D microstructures without steps on the surfaces, significantly improving the machining surface quality. The surface roughnesses were *R_a_* = 1.365 μm and *R_a_* = 1.51 μm, respectively.

## Figures and Tables

**Table 1:** Machining conditions for femtosecond laser cutting, EDM and thermal diffusion bonding.

Parameters	Femtosecond Laser Cutting	EDM	Thermal Diffusion Bonding
Central wavelength	800 nm	-	-
Laser power	400 mW	-	-
Cutting speed	100 μm/s	-	-
Pulse duration	35 fs	800 ns	-
Pulse interval	-	4200 ns	-
Pulse frequency	1 kHz	0.2 MHz	-
Machining voltage	-	90 V	-
Peak current	-	0.1 A	-
Bonding temperature	-	-	900, 950, 1000 °C
Bonding time	-	-	15 h
